# Supplementary material for: SIRT7 promotes genome integrity and modulates non‐homologous end joining DNA repair
Source: EMBO J. 2016 May 25;35(14):1488–503. doi: 10.15252/embj.201593499 (PMC4884211; doi:10.15252/embj.201593499)
Supplement: Supplementary file 1 — Appendix [file EMBJ-35-1488-s001.pdf]

Appendix Table of Contents

Appendix Figure Legends **2**
Appendix Figure S1 2
Appendix Figure S2 2
Appendix Figure S3 2
Appendix Figure S4 3
Appendix Figure S5 3
Appendix Figure S6 4
Appendix Figure S7 4
Appendix Figure S8 5
Appendix Supplementary Methods **7**
Appendix Supplementary Methods References 14
Appendix Figures **15**
Appendix Figure S1 15
Appendix Figure S2 16
Appendix Figure S3 17
Appendix Figure S4 18
Appendix Figure S5 19
Appendix Figure S6 20
Appendix Figure S7 21
Appendix Figure S8 22
Appendix Tables **23**
Appendix Table S1 23

Appendix Figure Legends

Appendix Figure S1.

A *SirT7*$^{-/-}$ mouse was generated by replacing exons 4 to 10 by a LacZ-Neomycin cassette.

B PCR of *SirT7* gene from tail DNA of WT, *SirT7*$^{+/-}$ and *SirT7*$^{-/-}$ mice.

C *SirT7* mRNA (left) and protein (right) levels from WT and *SirT7*$^{-/-}$ primary fibroblasts.

D Expected and observed Mendelian ratios from *SirT7* heterozygote cross in C57BL/6J mice (χ^2, $P <$ 0.0001).

E Weight distribution of WT and *SirT7*$^{-/-}$ male mice (n=3-10 male mice per time point and genotype; P value = 0.0258 by unpaired t-Test).

Appendix Figure S2.

A Growth curves for embryonic fibroblasts derived from 14.5d WT (n=4) and *SirT7*$^{-/-}$ (n=3) embryos cultured by passaging 10^6 cells per 100-mm dish every 3 days (3T3 protocol). The data is expressed as the mean ± SEM and is representative of two independent experiments using MEFs derived from multiple mouse litters.

B Quantitation of experiment shown in Figure 3A showing percentages of cells in G1, S and G2/M phase at passage 3 (P3) and 6 (P6) (n=3 per genotype). **P* < 0.05 by ANOVA Single Factor.

Appendix Figure S3.

A Representative images from neutral comet assays in passage 3 WT and *SirT7*$^{-/-}$ primary MEFs. DNA migrates proportionally to its degree of fragmentation (i.e. number of DSBs) which results in a comet-like shape.

B Western blot showing total levels of 53BP1 in WT and *SirT7*$^{-/-}$ cells before and after X-ray

irradiation (8Gy), and in the presence and absence of KU-55933 ATM inhibitor (ATM_i). ATM_i was added 30min prior to irradiation. One representative blot is shown from 4 independent experiments.

Appendix Figure S4.

A Cell cycle segmentation based on nuclear volume and EdU incorporation. Histogram shows the distribution of nuclear volume in EdU^+ (green) and EdU^- (blue) exponentially growing cells. Cells were pulsed with 10µM EdU for 30 minutes prior to fixation followed by EdU detection and counterstained with DAPI. Nuclei were imaged in 3D and segmented by DAPI staining followed by scoring for EdU incorporation. EdU^- cells display a bimodal distribution of nuclear volumes corresponding to 2N (G1, mean volume = 612μm^3) and 4N (G2, mean volume = 1203μm^3) DNA content. Outliers (polyploid or tiny cells) were excluded from further analysis. The central trough of EdU^- cells (red, dashed segment) was also excluded from downstream analysis as these cells are ambiguous as to their cell cycle state.

B Mean nuclear volumes in WT and *SirT7*$^{-/-}$ cells after application of the cell cycle segmentation strategy described in (A). Euchromatin volume (EC) is shown in blue, and heterochromatin (HC) in red. The sum of these two volumes gives the total nuclear volume. Segmented heterochromatin represents approximately 5% of the total nuclear volume. Euchromatin volume was estimated by subtracting heterochromatin from total nuclear volume. Note that the mean G2 volume is approximately double that of G1 cells and that there is no significant difference in the volumes of WT and *SirT7*$^{-/-}$ cells. Sample sizes: WT—G1=401, S=265, G2=77; *SirT7*$^{-/-}$—G1=224, S=231, G2=34.

Appendix Figure S5.

A, B IF analysis of passage 3 WT and *SirT7*$^{-/-}$ primary fibroblasts showing RAD51 dynamics after DNA damage induction. Cells were untreated or treated with 1Gy of X-Ray irradiation and fixed at the indicated times. 30 minutes prior fixation, cells were pulsed with EdU. Cells were then stained for RAD51 and then counterstained with DAPI (n>30 cells per group/mice; 3 mice per genotype). (A)

Representative image of WT and *SirT7*$^{-/-}$ cells (scale bar 5µm) showing Merge, γH2AX (red), Rad51 (green), and 3D rendering of reconstructed Z-stack with foci modeled as spheres (nucleus-pale blue, γH2AX-red spheres, Rad51-green spheres). (B) Quantitation of the described experiment described. Results are expressed as the mean ± SEM of 3 samples per genotype.

C Graph showing the relative contribution of repair pathways NHEJ (proxied by 53BP1) and HR (proxied by Rad51) in relation to the total damage load (proxied by γH2AX). Results are expressed as the mean ± SEM of 3 samples per genotype.

Appendix Figure S6.

A Western blot of cell lines used in (A), and in Figures 5H and 5I.

B Quantification of the GFP expression-based NHEJ repair reporter in *SirT7*-depleted HT1080 cells (SirT7 Knockdown) versus control (Scramble Control). Note that two individual SirT7 Knockdown cell lines have been used for the assay to exclude possible off-target effects.

C Representative dot plots showing DS-RED expression in HT1080 transfected with Scramble Control or SirT7 Knockdown.

D Quantitation of (D) showing percentages of cells DS-RED^{+} after 48h of transfection with 100ng of DS-RED vector. Results represent the mean ± SEM of 4 transfections per condition.

$^{**}P < 0.01$; $^{***}P < 0.001$; by ANOVA Single Factor.

Appendix Figure S7.

A-B NHEJ repair during class switching recombination. Lymphocyte development was measured after LPS and IL-4 treatment. (A) B cell proliferation measured by carboxyfluorescein succinimidyl ester (CFSE) dilution. (B) Flow cytometry quantitation showing percentage of IgG1^{+} cells in WT and *SirT7*$^{-/-}$ cells that have divided equally.

C-E SCE in WT and *SirT7*$^{-/-}$ B lymphocytes. Cells were isolated and cultured with LPS, IL-4 and BrdU for

48h. Next, metaphase spreads were prepared and SCE was analyzed. (C) Representative images of WT and *SirT7*$^{-/-}$ metaphase spreads. (D-E) Quantitation of (C) showing the exchanges per metaphase(D) and per chromosome (E). Results are expressed as the mean ± SEM of 3 samples per genotype. 24 to 50 metaphases were analyzed per sample.

F HR repair assay using a reporter locus stably integrated in HT1080 cells after overexpression of SIRT7 protein (SirT7-HA) or an empty vector. Relative HR activity is measured by cell colony formation after successful repair of an I-SceI-induced DSB within a puromycin resistance gene. Results are expressed as the mean ± SEM of triplicate transfections per condition.

G Western blots showing the efficiency of SIRT7 overexpression in the HR assay (F).

Appendix Figure S8.

A γH2AX accumulation after different microirradiation doses. HT1080 cells were irradiated over a 2 x 18 pixel area using a 405nm laser at 100% power for the indicated number of iterations and chased for the indicated times prior to fixation. Cells were stained for γH2AX (red) and counterstained with DAPI (blue). Note that 50 iterations produces a weak γH2AX signal indicative of partial DNA damage while 350 iterations produces a heavy γH2AX signal with additional γH2AX foci formation throughout the nucleus. The use of a 175 iteration dose produces a consistent medium intensity γH2AX strip, and most important, highly reproducible kinetics of SIRT7 recruitment to DNA damaged site (see (D)). 3 representative cells for each damage dose and time point are shown (scale bars 5 µm).

B SIRT7 accumulates at DSBs induced by laser microirradiation. HT1080 cells expressing HA tagged SIRT7 were microirradiated as in (A) at 175 iterations and allowed to rest for 30 minutes prior to fixation followed by staining for γH2AX (red), SIRT7-HA (green), and counterstained with DAPI (blue). 3 representative cells are shown. Scale bars 5 µm.

C SIRT7 is fully mobile in the nucleoplasm and partially immobilized in the nucleolus. FRAP analysis of HT1080 cells expressing *SirT7*-GFP in which a 1µm diameter spot within either the nuclear

compartment (red) or nucleolus (blue) was photobleached and monitored for fluorescence recovery for 2min at 0.5s intervals. Cells expressing GFP-only vector containing a SV40 nuclear localization signal were photobleached in the nuclear compartment (green) under the same conditions as control. FRAP analysis using HT1080 cells expressing SirT7-GFP indicated that SIRT7 localized within the nucleoplasm is completely mobile (full recovery of fluorescence intensity), whereas a fraction of nucleolar SIRT7 (16%) is immobile, in concurrence with many reports of SIRT7 interactions with ribosomal genes and nucleolar-associated proteins. (Sample sizes: *SirT7*-GFP Nucleus, n=12; *SirT7*-GFP Nucleolus, n=2; GFP-only, n=2)

D Kinetics of GFP-tagged SIRT7 to the sites of microirradiation-induced damage at different doses (50, 175 and 350 iterations). The relative fluorescence intensity of SIRT7-GFP was monitored at 5s intervals over 5min (mean ± SEM per time point; 50 iterations n=36; 175 iterations n=34; 350 iterations n=23). Note that a 50-iteration dose nearly fails to recruit SIRT7 at DNA damage sites, and 175- and 350-iteration doses recruits SIRT7 with similar kinetics but the 350 dose to a lesser extent, possibly due to an unbearable disruption of chromatin structure. Therefore, 175-iteration was used as the standard dose.

E Kinetics of GFP-tagged SIRT7 to the sites of DNA damage, as in Figure 6E. Data was acquired at 30s intervals over 30min (mean ± SEM; n=19). Note that at 30min post-insult we still observe 14% (±5.5% SEM) enrichment of SIRT7-GFP signal at the site of damage.

Appendix Supplementary Methods

Senescence associated β-Galactosidase activity

SA-βGal activity was performed according to manufacturer's instructions (Cell Signaling). Briefly, cells were washed once with PBS and fixed with 2% formaldehyde, 0.2% glutaraldehyde in PBS for 15min. Cells were washed again with PBS and stained with X-Gal solution (1mg/ml X-Gal, 40mM Citric acid, Sodium Phosphate, pH 6.0, 150mM NaCl, 2mM $MgCl_2$, 5mM Potassium Ferricyanide, 5mM Potassium Ferrocyanide) overnight at 37°C, and rinsed with PBS and methanol. Phase contrast images were acquired with a Zeiss Observer Z1 microscope equipped with LD Plan-Neofluar 20x objective lens and AxioCam MRc true-color camera.

GFP NHEJ reporter system

The plasmid containing a GFP reporter for NHEJ (Seluanov et al, 2004) was stably transfected in HT1080 cells, and clones containing a single copy of the reporter were identified by Q-PCR. *SirT7* knockdown and control cells in this background were further generated by stably transfecting -shRNA-SirT7 and shRNA-Scr plasmids. After 10 days of selection with 1 µg/ml Puromycin (Invitrogen), the pool of transfected cells was further transfected with the pCMV-I-SceI plasmid. Cells were harvested 72 hours post-transfection and subjected to FACS analysis to determine percentage of GFP positive cells.

HR reporter assay

The Homologous Recombination (HR) reporter locus comprises two defective puromycin genes configured as an inverted repeat where HR activity restores puromycin resistance (Lio et al, 2004). HR repair was induced in HT1080 cells by transient expression of I-SceI endonuclease. Cells were plated into 6-well trays at 300,000 cells/well, and transfected the next day with pCMV-I-SceI together with pcDNA4T0-SIRT7-HA or pcDNA4To empty vector using LT1 (Mirus). 24 h after transfection, cells were re-plated at 50,000 cells/10 cm dish for colony formation under selection with puromycin (1.0 µg/ml). To

measure cloning efficiency, non-selected cells were plated at 250 cells/10 cm dish in triplicate. Colonies were stained and counted after 9 days. The frequency of HR repair is calculated as the average number of colonies/dish normalized by cloning efficiency.

Random integration assay

The random integration of foreign DNA and the I-SceI-GFP reporter were used to measure NHEJ repair as described previously (Yeung et al, 2012), with some modifications. HT1080 cells were transfected with 1.5µg of an EcoRI-linearized peGFP-C3 vector together with 1.5µg of either: pcDNA4-SirT7, pcDNA4-empty, shRNA-SirT7-pRS or shRNA-Scr-pRS plasmids. 48 hours later, $2.5x10^4$ cells/10 cm culture dish were plated in triplicate for G418 selection. To measure cloning efficiency, cells were also plated in triplicate at 250 cells/10 cm dish. One day after, G418 (Invivogen) was added at a final concentration of 1mg/ml. Colonies were counted after 10 days. To measure the effect of H3 mutants, H1080 cells were transfected with an EcoRI-linearized pMSCV vector with pCMV6-WTH3, H3-K18Q or H3-K18R mutants. 48 hours later, $2x10^5$ cells were plated in triplicate for selection. One day after, Puromycin (Invitrogen) was added at final concentration of 1µg/ml. Plating efficiency was analyzed as described above.

ChIP-at-a-break HT1904 cells containing a single I-SceI site within the puromycin acetyltransferase (puromycin) gene were previously described (Fnu et al, 2011). . To induce the I-SceI-dependent DSB, $1x10^7$ *SirT7* KD and control cells were resuspended in 250µl of Ingenio buffer (Mirus) and were electroporated with 15µg of pCMV-I-SceI or ISceI-GR-RFP plasmid using a BioRad device (Gene Pulser Xcell) with 4mm gap cuvette (BioRad) at 200V and 950µF. Electroporated cells were then transferred to complete medium in the presence or absence of 10µM PARP1/2 inhibitor, Olaparib, and incubated at 37°C for 24 hours. Following I-SceI expression, cells were harvested for ChIP. First, cells were fixed for 15 minutes with 1% formaldehyde, washed twice with PBS, and then collected from dishes. Cells were centrifuged and supernatant discarded. For SIRT7 and 53BP1, ChIP was performed on nuclear extracts.

First, cells were ressuspended in Lysis Buffer 1 (5 mM HEPES, 85 mM KCl, 0.5% NP-40) to disrupted cellular membrane and incubated for 5 minutes on ice. After centrifugation, nuclei were ressupended in Lysis Buffer 2 (1% SDS, 10mM EDTA, 50mM Tris 50mM and protease inhibitors) and incubated on ice for 40minutes. For H3K18c ChIP, whole cell extracts were obtained by directly lysing cells in Lysis Buffer 2 for 40 min on ice. Chromatin was then sonicated to an average fragment size of 300bp. Samples were then centrifuged at 12,000 RPM, 10°C for 15min and supernatants were collected. For the ChIP procedure, protein-A and protein-G coupled magnetic beads (Dynabeads, Life technologies) were first blocked with 1% BSA in PBS for 30min at 4°C on a rotator. To conjugate the anti-H3K18Ac antibody to the beads, 300μl of 1% BSA in PBS containing 10μg of antibody was incubated with the beads for 4h, followed by 3 washes of 1% BSA in PBS and 1h of blocking with 1% BSA prior to the addition of chromatin samples. After measuring DNA concentration and sonication efficiency, sonicated lysates were diluted with Dilution Buffer (1% Triton X-100, 2mM EDTA, 20mM Tris-HCl, pH 8.0, 150mM NaCl and protease inhibitors) and were incubated overnight at 4°C on a rotator. Beads were then washed 5 times with ice cold RIPA buffer (1% NP-40, 0.7% Na deoxycholate, 50mM Tris, pH 8.0, 1mM EDTA, 500mM $LiCl_2$) and once with modified TE (0.1mM EDTA, 10mM Tris-HCl). Reversal of crosslinks was performed with 100μl of RC buffer (0.1M $NaHCO_3$, 1% SDS) for 6h at 60°C followed by purification using a standard PCR purification kit (Quiagen). Concentration of ChIP and INPUT DNA was measured using PicoGreen (Life Technologies). Quantitative PCR was performed using 1ng of ChIP and INPUT DNA. Samples were normalized to INPUT and expressed as a ratio relative to non-I-SceI treated cells. See Supplemental Table 1 for primer sequences.

Western blot assays

To harvest whole cell extracts, cells were resuspended in Lysis Buffer (50mM Tris-HCl, pH 7.5, 200mM NaCl, 1% Tween-20, 0.2% Igepal, 50mM Glycerol Phosphate, and protease inhibitors) and incubated on ice for 40 min. Samples were then centrifuged and supernatants collected. For chromatin and

nucleoplasmic fractions, cells were lysed with Buffer A (10mM Tris-HCl, pH 7.9, 1.5mM $MgCl_2$, 10mM KCl, 0.1mM PMSF, 0.5mM DTT, and protease inhibitors). After centrifugation at 13,000 RPM for 30s, the nuclear pellet was resuspended in Buffer C (10mM Tris-HCl, pH 7.9, 1.5mM $MgCl_2$, 0.42M NaCl, 25% glycerol, 0.2mM EDTA, 0.1mM PMSF, 0.5mM DTT, and protease inhibitors) and left for 20min on ice. The samples were centrifuged at 7,000G for 10min. The supernatant containing the nucleoplasmic fraction was combined with 5x Laemmli Sample Buffer, whereas the pellet containing the chromatin fraction was re-suspended in 1x Laemmli buffer and then sonicated. Protein concentration was determined with BCA Protein Assay kit (Pierce) using BSA as a standard. Western blot analysis was performed according to standard procedures using chemiluminescence detection (Santa Cruz).

Immunofluorescence

IF and FISH assays were carried out as previously described (Serrano et al, 2013). Briefly, images were acquired using a Zeiss LSM510 Meta confocal microscope equipped with a C. Apochromat 63x/1.2 NA water objective. For foci quantification experiments, images were deconvolved with Huygens software (SVI, NL) using an empirically determined PSF. In all experiments, images were corrected for chromatic shift in the x, y, and z axes by intra-experimental acquisition and analysis of TetraSpeck microspheres (Invitrogen). 3D-reconstruction and image analysis were performed using Imaris software (Bitplane, A.G.). Nuclei and heterochromatin were segmented based on DAPI staining intensity using the standard Surfaces function of Imaris. Heterochromatin (i.e. pericentric heterochromatin or "chromocenters") was adaptively segmented based on per-nucleus DAPI intensity using the following formula to determine segmentation threshold: *Threshold = (Nuclear DAPI Intensity Mean) + (1.5 × Nuclear DAPI Intensity Standard Deviation)*. Foci were detected and quantified using the standard Spots function of Imaris in region growing mode to allow for different foci sizes. Foci seeds were detected and segmented based on "quality" value, and allowed to grow to a determined intensity threshold. All images amongst all samples within a given experiment were acquired and quantified using the same microscope and

analysis parameters. Cell cycle was determined in exponentially growing cells as previously described (Serrano et al, 2011). Briefly, exponentially growing cells were pulse labeled with 10µM EdU (Invitrogen) 30min prior to fixation. After nuclear segmentation, the 3D nuclear volumes of EdU^+ and EdU^- cells were plotted in a histogram. EdU^- cells demonstrate a bimodal distribution corresponding to 2N (G1) and 4N (G2) DNA content, while outliers (polyploid cells) and the central trough (ambiguous cells) were discarded from further analysis (see Figure S4 for more details).

Laser microirradiation and live cell imaging

Microirradiation experiments and quantification of recruitment kinetics was performed following the methods described (Hable et al, 2012) with some modifications. FRAP experiments were carried out as previously described (Bosch-Presegue et al, 2011) (see Figure S8 for more details). Live cell imaging, microirradiation, and photobleaching (FRAP) experiments were carried out with a Zeiss 510 META confocal laser scanning microscope equipped with a C. Apochromat 63x/1.2 water objective and a heated environmental chamber set to 37°C with 5% CO_2 and 95% relative humidity. Confocal image time series were recorded with a frame size of 512 x 512. For microirradiation experiments, HT1080 cells were sensitized to DNA damage with 10µM 5-Iodo-2'-deoxyuridine (IdU, Sigma) 24h prior to each experiment. DNA damage was induced using a 405nm diode laser set to 100% transmission, and focused on a 2µm diameter spot within the nucleus, but outside of any nucleoli. Fluorescence intensities at the site of damage induction and within the nucleolus were monitored at 5s second intervals for five minutes. For evaluation of recruitment kinetics, fluorescence intensities of the irradiated regions were corrected for background and photobleaching, and then normalized to the pre-irradiation intensity value. Quantification and curve fitting followed the methods described in (Hable et al, 2012) using the double exponential function described (eq. 1) which allows quantification of both accumulation and decay dynamics of protein recruitment. To model the depletion kinetics of the nucleoli, the double exponential model was modified to invert the exponents, as well as swapping τ_1 and τ_2, to allow for

decay-accumulation kinetics rather than accumulation-decay kinetics. FRAP experiments were carried out as previously described (Bosch-Presegue et al, 2011). Briefly, HT1080 cells expressing SirT7-GFP or a GFP vector containing a SV40 NLS signal (Invitrogen) were subjected to photobleaching of a 2 µm diameter spot using the 488nm laser line from a 30mW Argon laser at 100% power intensity over 50 iterations (128µs duration), and fluorescence recovery observed over a two minute period at 500ms intervals. Fluorescence levels within the ROI were double normalized to the background intensity as well as a reference ROI to account for photobleaching effects. Data was modeled using IgorPro 6.1 (WaveMetrics, Lake Oswego, OR, USA) software with a FRAP calculation macro (K_FRAPcalcV9.ipf, Kota Miura, EMBL, Germany), yielding half-life of recovery and immobile fraction datum.

Flow cytometry

Cell cycle and polyploidy analysis was performed as previously described (Serrano et al, 2013). For cell survival analysis, thymocytes were stained with Annexin V-FITC (Ebiosciences) and 7AAD (Pharmigen) using standard methods. For class switching, splenic B cells were stained with anti-B220-FITC and anti-IgG1-APC. For analysis of HSCs, BM cells were stained with antibodies against the following surface markers: CD3–APC, CD11b–APC, CD45R (B220)–APC, Ter119–APC, Gr-1–APC, Sca1–FITC, CD117 (c-Kit)-PE. Lineage markers CD3, CD19, B220, Gr1, Mac1, and Ter119 were used for the exclusion of lineage positive cells. HSC was further defined as lin^{-}Sca^{+}c-kit^{+}. For HSC and class switching, anti-CD16/CD32 antibodies were also used to avoid nonspecific antibody binding. All samples were run with a FACScalibur or a FACS FC500 (Beckman Coulter) and data was analyzed with Flow Jo 7/8 (Tree Star).

RNA isolation and quantitative PCR

Total RNA was isolated using Trizol reagent (Life Technologies) according to manufacturer’s instructions. cDNA was generated with the TaqMan Gold RT-PCR kit (Applied Biosystems) using 1µg of RNA as input.

Quantitative PCR was performed with SYBR Green (Life Technologies) and the ABI 7900HT Sequence Detection System. See Supplemental Table 1 for primer sequences.

Sister chromatid exchange

B cells were isolated and cultured with 10μM BrdU for 48h. Metaphase spreads were prepared as described elsewhere and were dropped onto slides (Misenko & Bunting, 2014). Slides were then immersed in 2xSSC for 5min, incubated with 10μg/μl Hoechst 33258 for 20min and rinsed with Maclavaine solution for 10min. Slides were then UV irradiated with a transilluminator for 45min and incubated with 1xSSC at 55°C for 45min. After 30min incubation in ddH2O, slides were then stained with 8% GIEMSA solution for 30min, rinsed with water and allowed to air dry. Finally, slides were immersed in Xylene for 15s and mounted. Images were obtained with a Zeiss AxioImager Z2 microscope equipped with a Metasystems Metafer Automated Slide Scanning Platform.

Antibodies

The following antibodies were used in this study: anti-actin (Abcam, ab2287), anti-Histone H3 (Abcam, ab1791), anti-pATM (Ser1981; BD Pharmingen, 560007), anti-ATM (Cell Signaling, 2873S), anti-pKAP1 (Ser824; Bethyl, A300-767A), anti-KAP1 (Abcam, ab109287), anti-53BP1 (for WB, Bethyl, A300-272A; For IF, Novus, NB100-304), anti-Rad51 (Santa Cruz, sc-8349), anti-γH2AX (Millipore, 05-636), anti-H3K18Ac (Abcam, ab1191), anti-H4K20me2 (Active Motif, 39173), anti-HA (Sigma, H6908), Anti-Flag (Sigma, F1804), anti-Myc-Tag (Cell Signaling, 2276), anti-SIRT7 (Cell Signaling, 5360), anti-MDC1 (Abcam, ab11169), anti-H2A (Cell Signaling, D603A), anti-K63 linked ubiquitin (Abcam, ab179434), anti-GAPDH-HRP (Abcam, ab9482), anti-rabbit-HRP (Santa Cruz, sc-2077), anti-mouse-HRP (Santa Cruz, sc-2314), anti-Sca1-FITC (eBioscience, 11-5981-81), anti-c-kit-PE (eBioscience, 12-1171081), anti-IgG1-biotin (BD Pharmingen, 553441), anti-B220-PeCy5 (eBioscience, 46-0452-80), anti-CD8-PE (eBioscience, 12-0081-81), anti-CD4-FITC (eBioscience, 12-0043-82), anti-CD45.2-APC (eBioscience, 17-0454-81), anti-

CD16/CD32 (eBioscience, 14-0161-82) and SA-APC (eBioscience, 17-4317-82). A biotin Mouse Lineage Panel (BD Pharmingen, 559971) was also used. The anti-RIF1 antibody was a kind gift from Dr.Buonomo. Anti-SIRT7 antibody was obtained from BiosChile S.A.

Appendix Supplementary Methods References

Bosch-Presegue L, Raurell-Vila H, Marazuela-Duque A, Kane-Goldsmith N, Valle A, Oliver J, Serrano L, Vaquero A (2011) Stabilization of Suv39H1 by SirT1 is part of oxidative stress response and ensures genome protection. *Mol Cell* **42:** 210-223

Fnu S, Williamson EA, De Haro LP, Brenneman M, Wray J, Shaheen M, Radhakrishnan K, Lee SH, Nickoloff JA, Hromas R (2011) Methylation of histone H3 lysine 36 enhances DNA repair by nonhomologous end-joining. *Proc Natl Acad Sci U S A* **108:** 540-545

Hable V, Drexler GA, Bruning T, Burgdorf C, Greubel C, Derer A, Seel J, Strickfaden H, Cremer T, Friedl AA, Dollinger G (2012) Recruitment kinetics of DNA repair proteins Mdc1 and Rad52 but not 53BP1 depend on damage complexity. *PLoS One* **7:** e41943

Lio YC, Schild D, Brenneman MA, Redpath JL, Chen DJ (2004) Human Rad51C deficiency destabilizes XRCC3, impairs recombination, and radiosensitizes S/G2-phase cells. *J Biol Chem* **279:** 42313-42320

Misenko SM, Bunting SF (2014) Rapid analysis of chromosome aberrations in mouse B lymphocytes by PNA-FISH. *J Vis Exp*

Seluanov A, Mittelman D, Pereira-Smith OM, Wilson JH, Gorbunova V (2004) DNA end joining becomes less efficient and more error-prone during cellular senescence. *Proc Natl Acad Sci U S A* **101:** 7624-7629

Serrano L, Liang L, Chang Y, Deng L, Maulion C, Nguyen S, Tischfield JA (2011) Homologous recombination conserves DNA sequence integrity throughout the cell cycle in embryonic stem cells. *Stem Cells Dev* **20:** 363-374

Serrano L, Martinez-Redondo P, Marazuela-Duque A, Vazquez BN, Dooley SJ, Voigt P, Beck DB, Kane-Goldsmith N, Tong Q, Rabanal RM, Fondevila D, Munoz P, Kruger M, Tischfield JA, Vaquero A (2013) The tumor suppressor SirT2 regulates cell cycle progression and genome stability by modulating the mitotic deposition of H4K20 methylation. *Genes Dev* **27:** 639-653

Yeung PL, Denissova NG, Nasello C, Hakhverdyan Z, Chen JD, Brenneman MA (2012) Promyelocytic leukemia nuclear bodies support a late step in DNA double-strand break repair by homologous recombination. *J Cell Biochem* **113:** 1787-1799

Appendix Figure S1

A

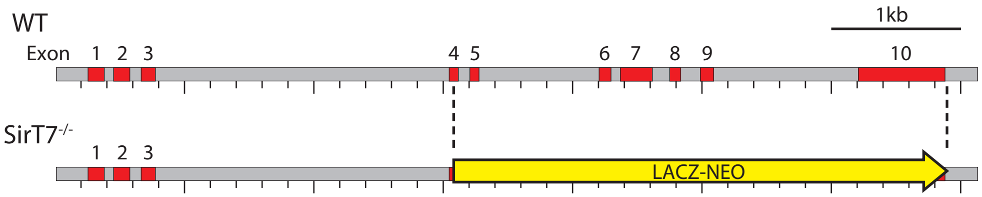

B

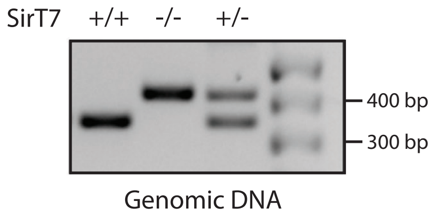

C

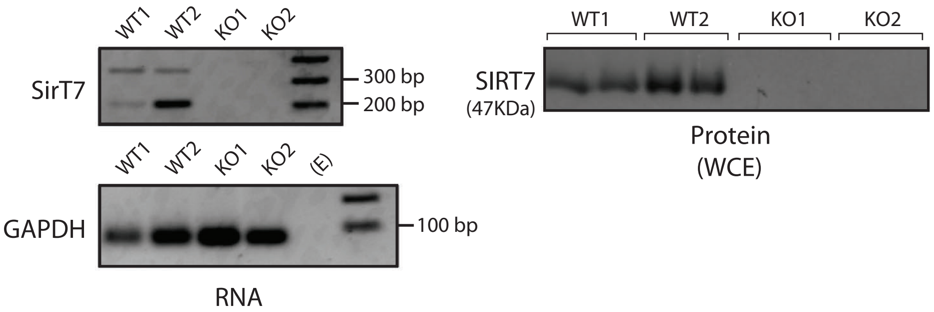

D

SirT7 genotype	+/+	+/-	-/-
Expected	25%	50%	25%
Observed pups	32% (n=58)	64% (n=116)	4% (n=8)

E

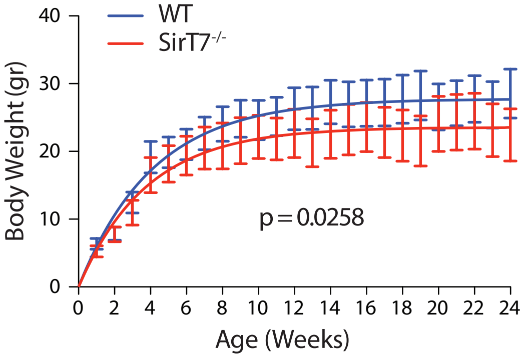

Appendix Figure S2

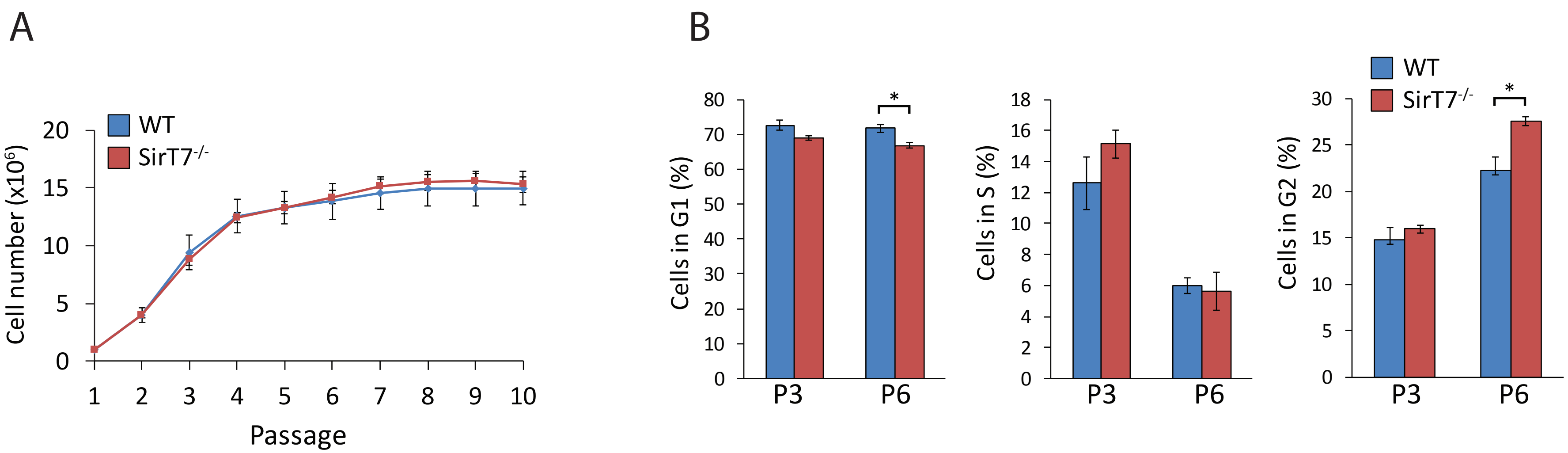

Appendix Figure S3

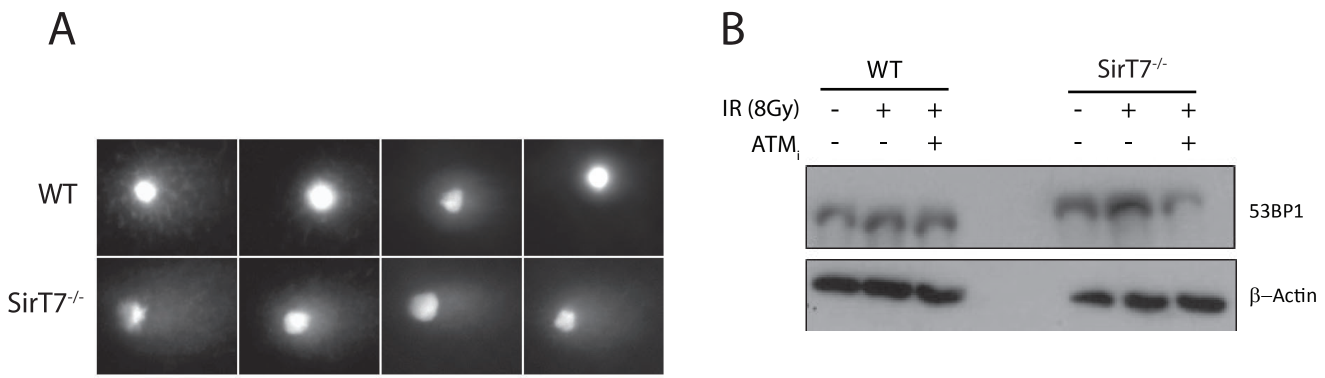

Appendix Figure S4

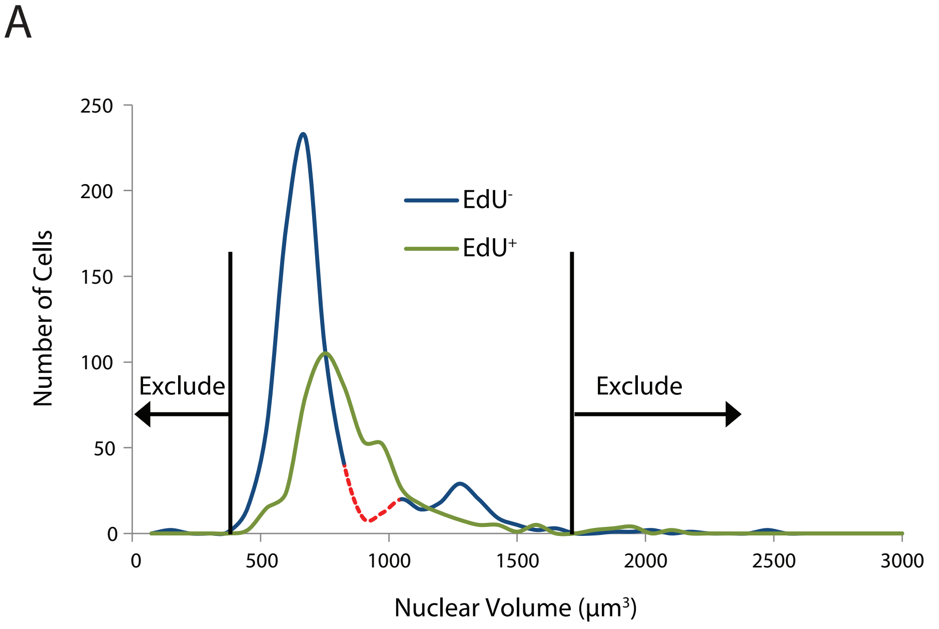

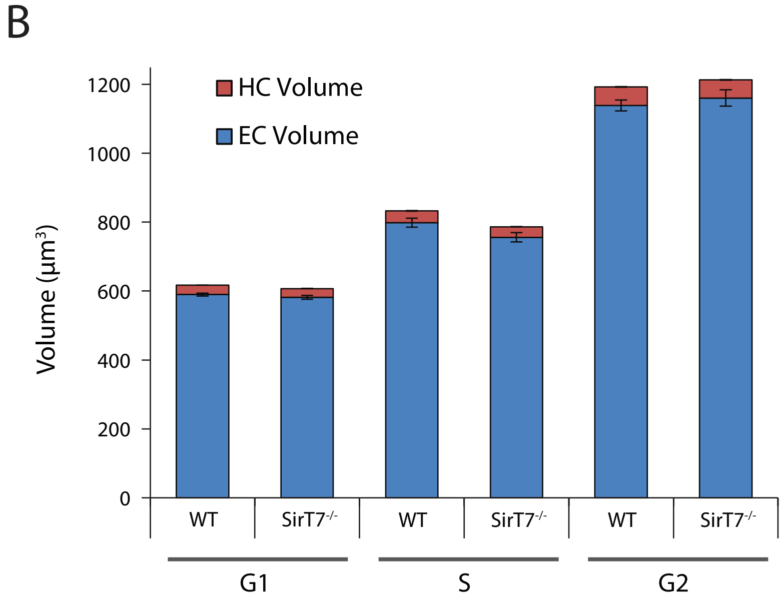

Appendix Figure S5

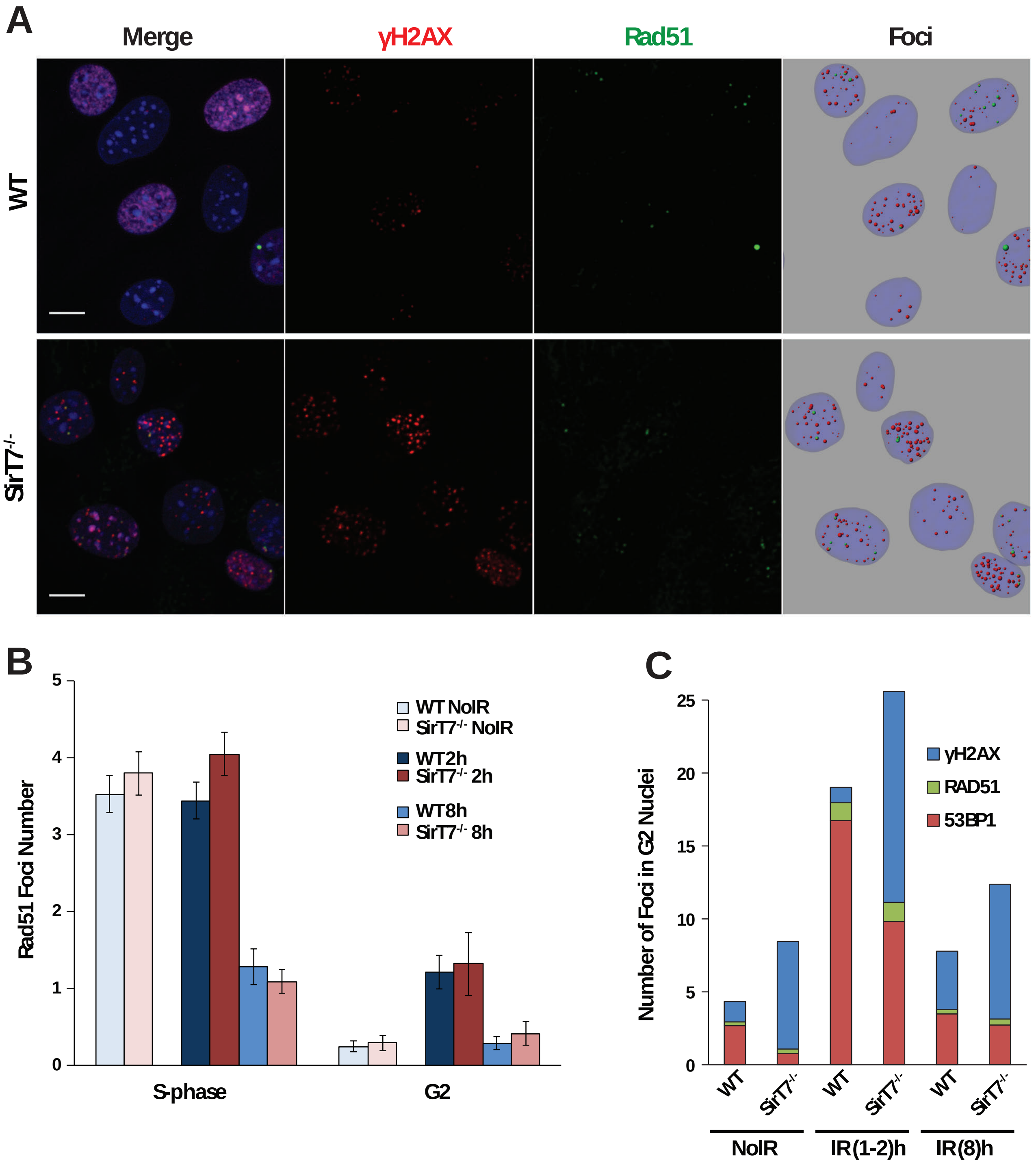

Appendix Figure S6

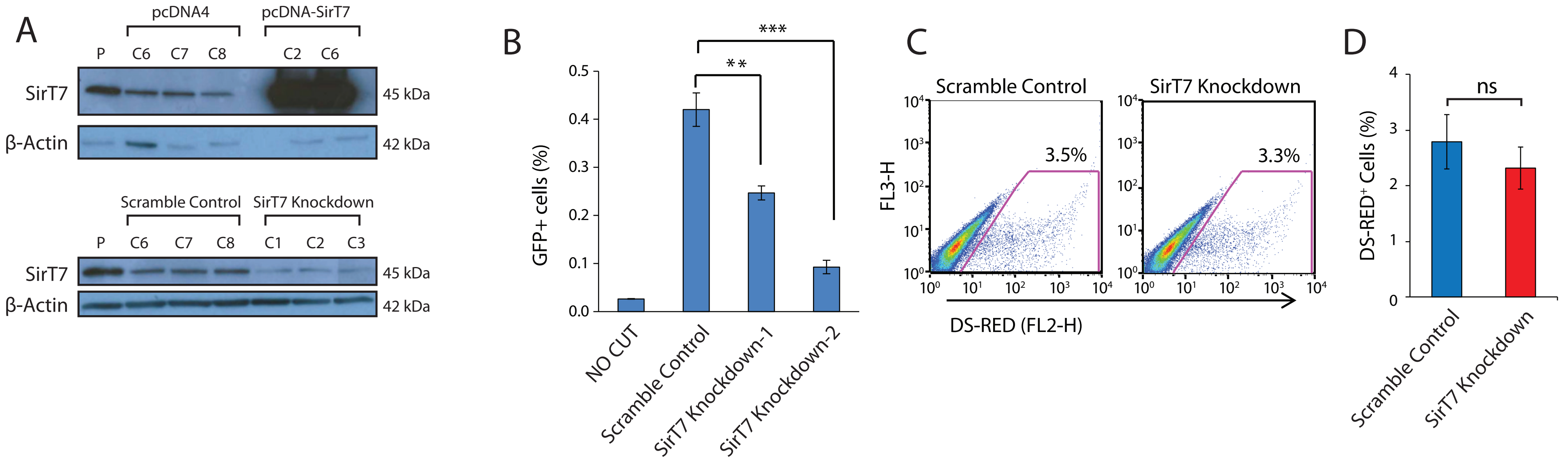

Appendix Figure S7

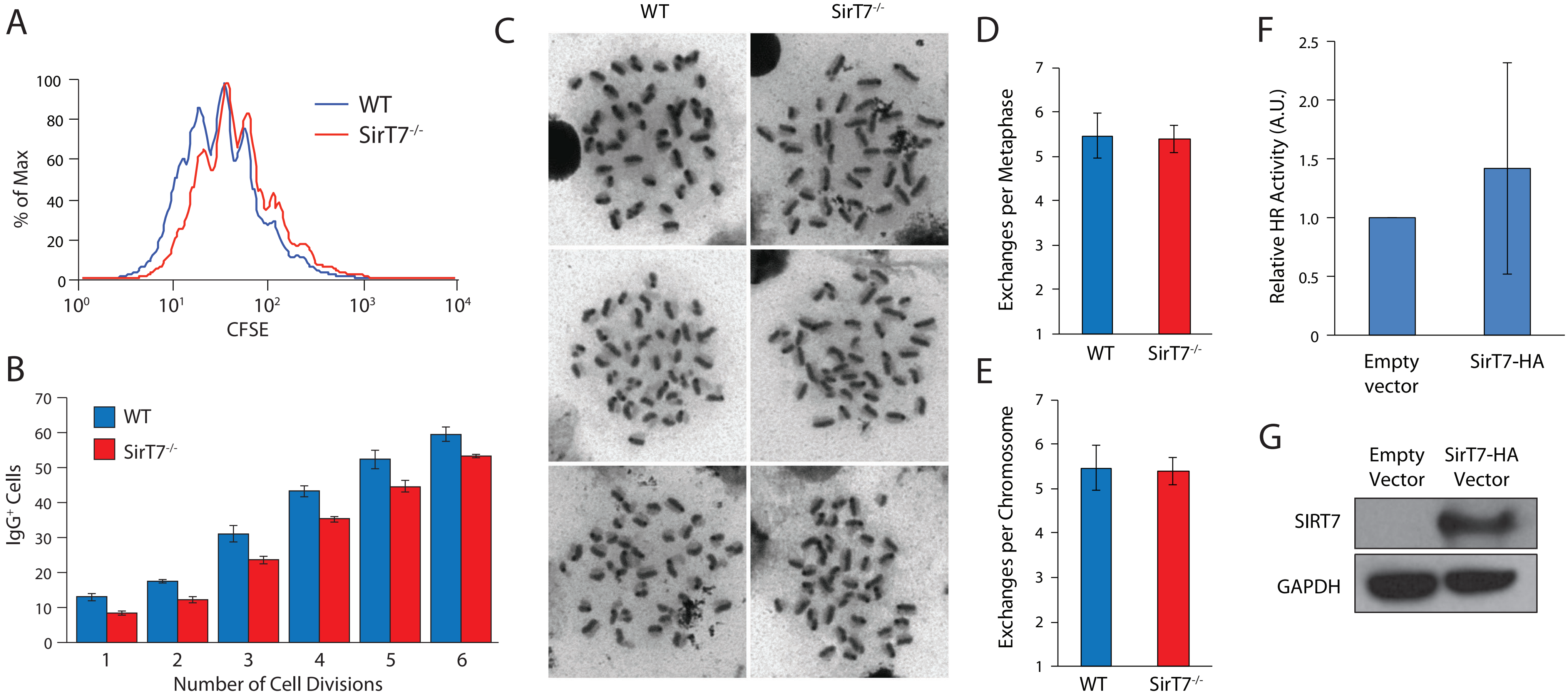

Appendix Figure S8

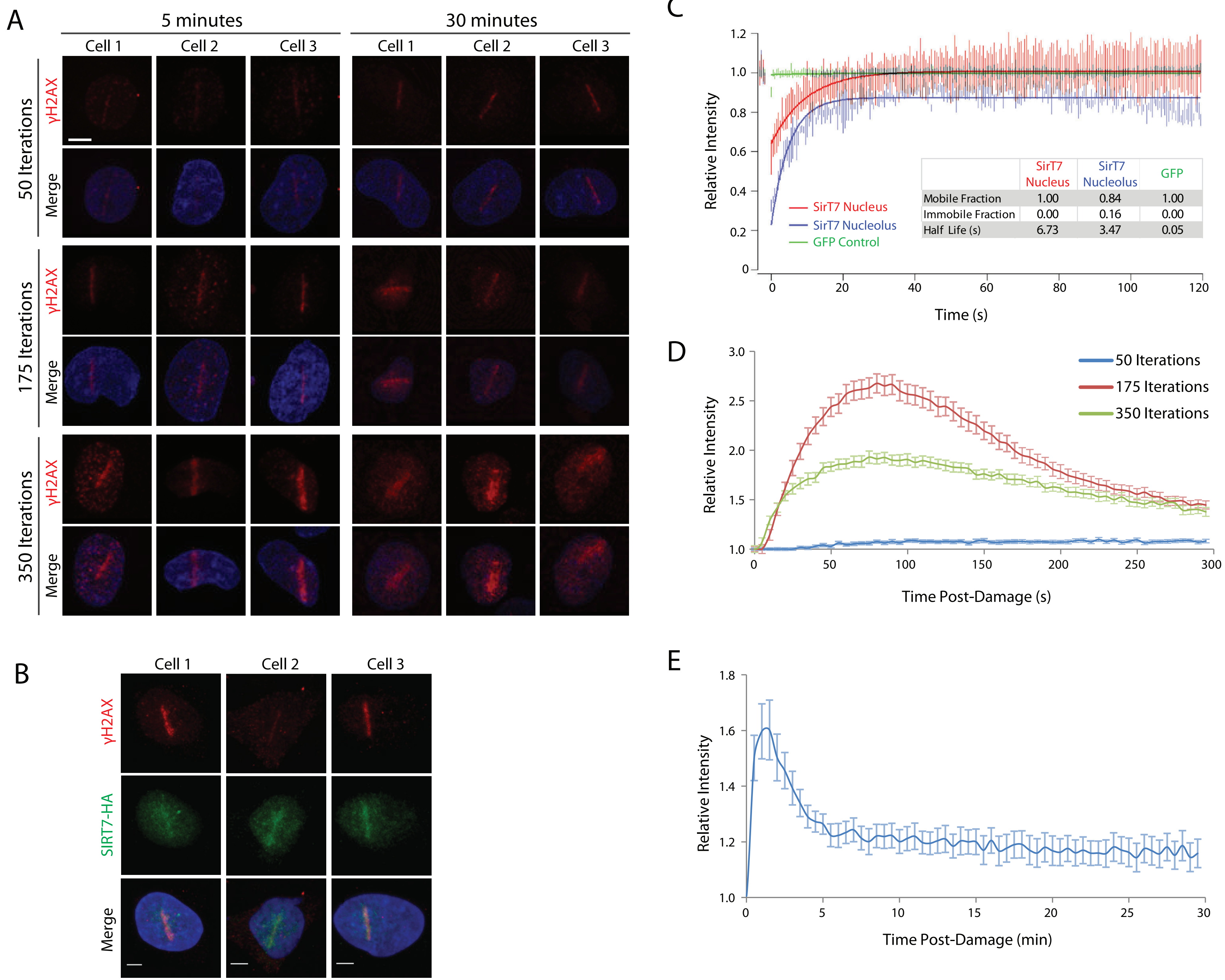

Appendix Table S1: List of primers used in this study.

Primer name	Forward Sequence (5'-3')	Reverse Sequence (5'-3')	Application
**SirT7**	GACCAGTTGTCCCCTTCT G	CACAGGCCTTCCTTTCTGAAG<br>GTCTGTCCTAGCTTCCTCACTG	Genotyping
**Aprt**	ATAAGACCCTGCCCTTCCTCTACA	TGCCTGCTTGCCGAATATCATGGT<br>ACAACCTTCCCTCCTTACCCTAACAG	Genotyping
**K18Q**	GGCAAGGCGCCACGCCAGCAGTTGGCCACTAAG	CTTAGTGGCCAACTGCTGGCGTGGCGCCTTGCC	Mutagenesis
**K18R**	CAAGGCGCCACGCAGACAGTTGGCCAC	GTGGCCAACTGTCTGCGTGGCGCCTTG	Mutagenesis
**SirT7-H118Y**	ATGTACATGTTCCCGTAGAG CTCGGAGATGG	CCATCTCCGAGCTCTACGGGAACATGTACAT	Mutagenesis
**+56 to +248**	GTCACCGAGCTGCAAGAACT	CAGGAGGCCTTCCATCTGT	Chip-at-the break
**+680 to +832**	CTTTTGAAGCGTGCAGAATG	GCTTTGCTCCTTCGCTTTC	Chip-at-the break
**+4054 to +4142**	TTTACTGGGGGACCTTGTG	GATGCCCCTGTTCTCATTTC	Chip-at-the break
**P16**	GTACCCCGATTCAGGTGATG	GGAGAAGGTAGTGGGGTCCT	qPCR
**SirT7**	AGAACTGTGATGGGCTCCAC	TGAAGGGCAGTACGCTCAGT	qPCR
**GAPDH**	ATGACATCAAGAAGGTGGTGAAG	TCCTTGGAGGCCATGTAGG	qPCR
