# Supplementary material for: SIRT7 promotes genome integrity and modulates non‐homologous end joining DNA repair
Source: EMBO J. 2016 May 25;35(14):1488–503. doi: 10.15252/embj.201593499 (PMC4884211; doi:10.15252/embj.201593499)
Supplement: Supplementary file 2 — Expanded View Figures PDF [file EMBJ-35-1488-s002.pdf]

Expanded View Figures

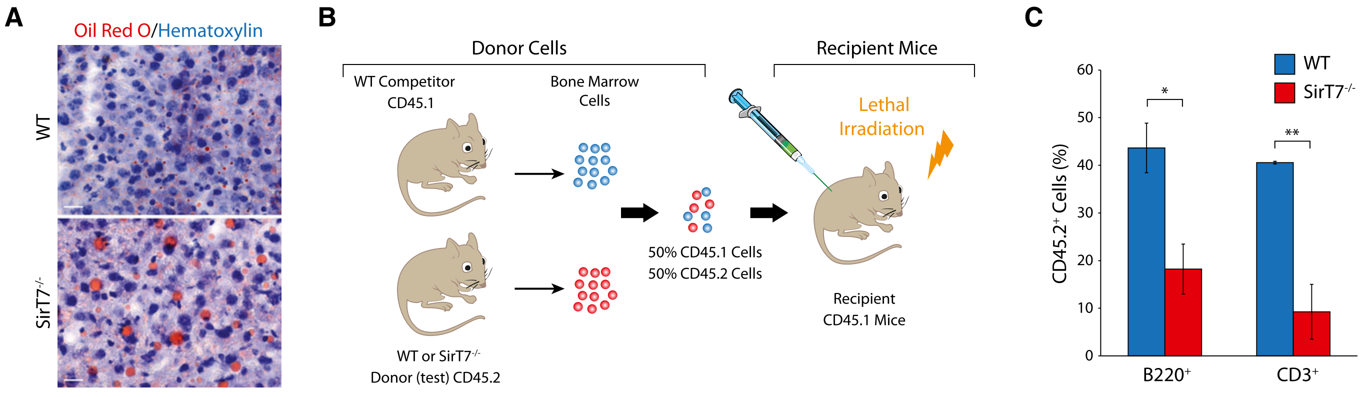

Figure EV1. Accelerated aging phenotype in *SirT7*$^{-/-}$ mice.

A Representative image of liver cryosections from 3-month-old WT and *SirT7*$^{-/-}$ mice stained with Oil Red O (red) and counterstained with hematoxylin (blue) (n = 3 livers per genotype). Scale bar 20 µm.

B Schematic representation of competitive bone marrow transplants, where equal numbers of WT or *SirT7*$^{-/-}$ bone marrow cells (CD45.2) were mixed with WT competitor cells (CD45.1) and transferred into lethally irradiated recipients via injection into the tail vein.

C Graph showing the reconstitution of the lymphoid compartment evaluated in peripheral blood by FACS using the CD45.2 marker 8 weeks later (mean ± SEM; 5 mice per genotype from two independent experiments). *P < 0.05; **P < 0.01; by ANOVA single factor.

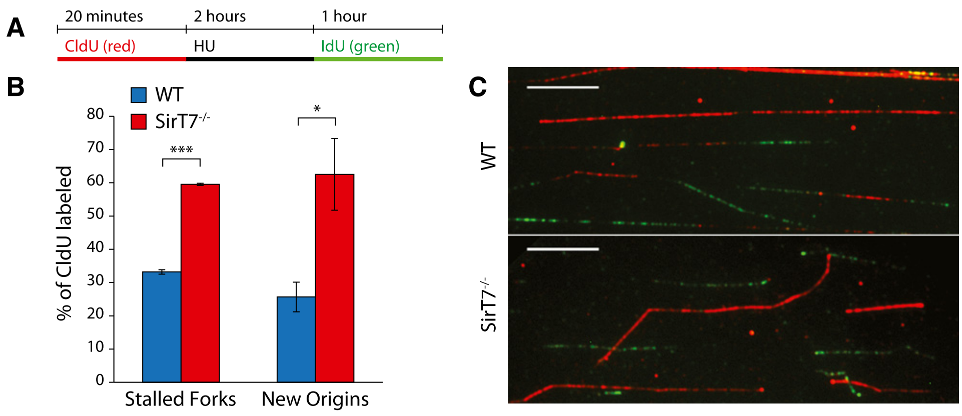

Figure EV2. Increased replication fork stall and new origin firing in *SirT7*$^{-/-}$ MEFs.

A Schematic representation of labeling protocol used in (B, C). Cells were pulse-labeled with CldU (red) for 20 min, treated with 2 mM HU for 2 h, and released into media containing IdU (green) for 1 h.

B Quantitation of the experiment depicted in (A, C), using WT and *SirT7*$^{-/-}$ primary MEFs in passage 3. Data represent the relative number of stalled forks (CldU only, red), and new origins (IdU only, green) from the total number of replication tracks labeled with CldU (mean ± SEM; three samples per genotype). *P < 0.05; **P < 0.01; ***P < 0.001 by ANOVA single factor.

C Representative images from the experiment described in (A) and quantified in (B). Scale bar 10 µm.

© 2016 The Authors

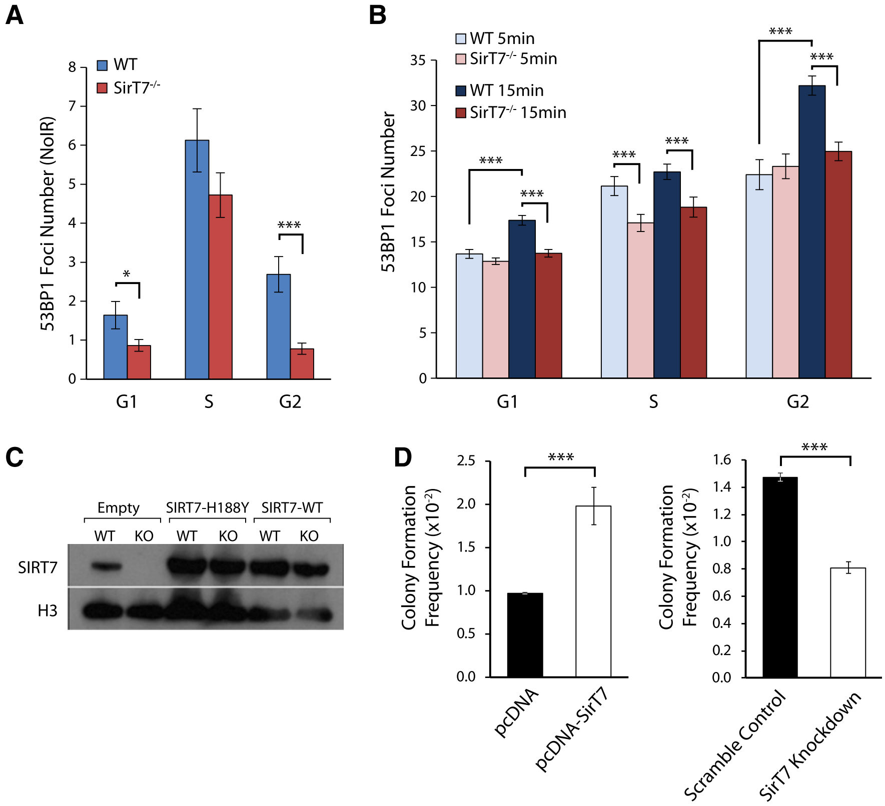

Figure EV3. Impaired NHEJ DNA repair pathway in *SirT7*$^{-/-}$ cells.

A, B IF of 53BP1 foci in cells from WT and *SirT7*$^{-/-}$ fibroblasts during the cell cycle in untreated (NoIR) conditions (A) or the indicated time points after IR (1 Gy). (B) Cells were pulsed with EdU, stained with antibodies against γH2AX and 53BP1, and then counterstained with DAPI. Data represent the quantitation of the number of 53BP1 foci throughout the cell cycle ($n > 30$ cells per group/mice; 3 mice per genotype; mean ± SEM).

C Western blot showing levels of SIRT7 protein from Fig 5D and E. Histone H3 was probed as a loading control.

D NHEJ repair assay using random integration of a linearized plasmid that confers G418 antibiotic resistance. Data show relative NHEJ activity by colony formation in SIRT7-overexpressing or SIRT7-deficient HT1080 cells, and their respective controls: empty vector (pcDNA) and scramble control (mean ± SEM of triplicate transfections. One representative experiment of two is shown).

Data information: *$P < 0.05$; **$P < 0.01$; ***$P < 0.001$ by ANOVA single factor.

Source data are available online for this figure.

 © 2016 The Authors

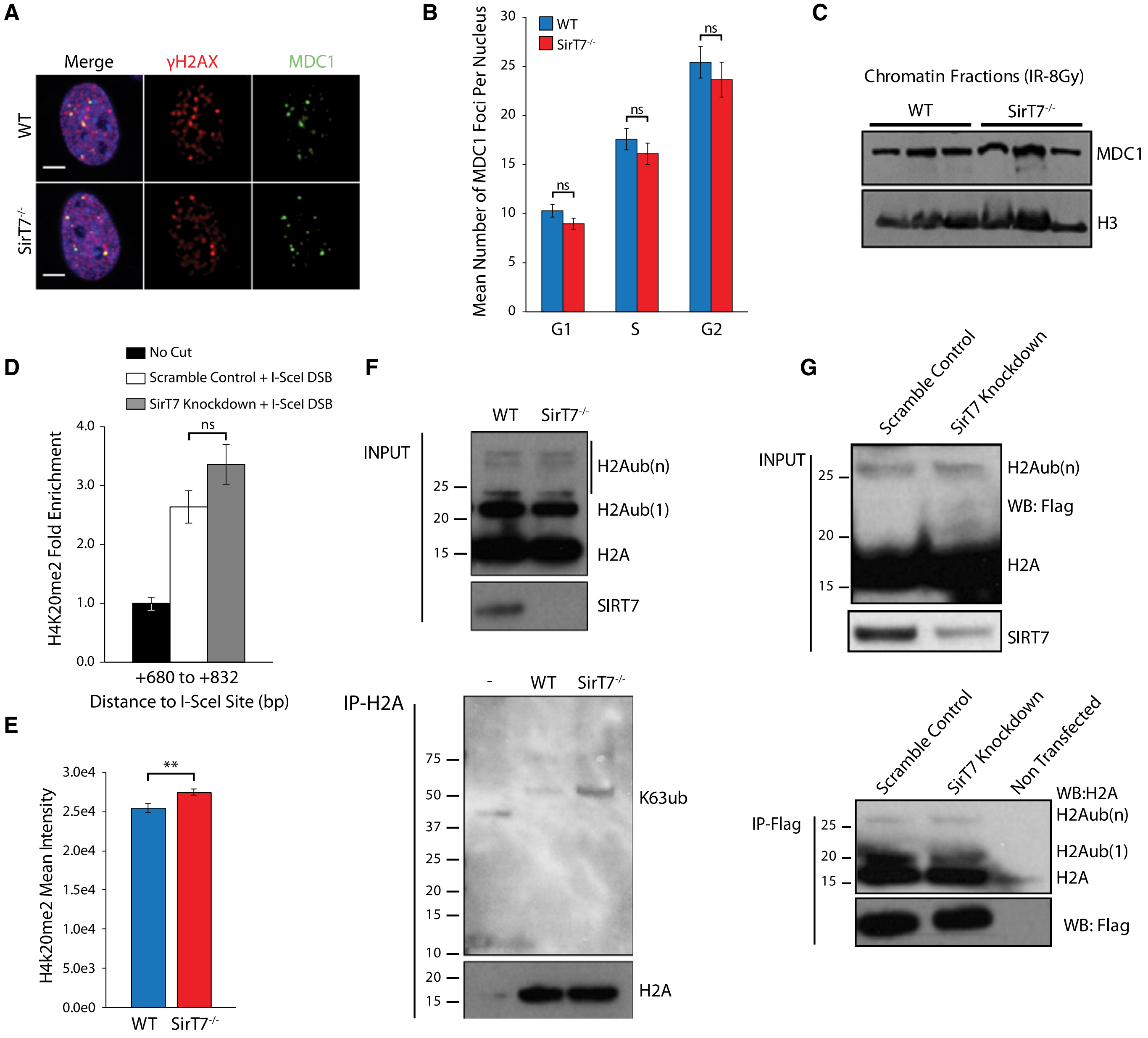

Figure EV4. Chromatin pathways upstream of 53BP1 appear unaffected in *SirT7*$^{-/-}$ cells.

A, B IF analysis of passage 3 WT and *SirT7*$^{-/-}$ primary fibroblasts showing MDC1 foci formation. Cells were IR (1 Gy), pulsed with EdU, and fixed after 1 h. Cells were then stained for MDC1, γH2AX, and then counterstained with DAPI ($n > 30$ cells per group/mice; 3 mice per genotype). (A) Representative image of WT and *SirT7*$^{-/-}$ cells (scale bar 5 μm) showing merge, γH2AX (red), and MDC1 (green). (B) Quantitation of the experiment in (A) showing the mean number of MDC1 foci per nucleus in each cell cycle stage.

C Western blot analysis for MDC1 in whole-cell extracts from WT and *SirT7*$^{-/-}$ cells before and after IR (20 Gy).

D H4K20me2 ChIP-on-break assay as described in Fig 5H (mean ± SEM; one of two independent experiments is shown).

E IF analysis of cells from WT and *SirT7*$^{-/-}$ fibroblasts, stained for H4K20Me2 and counterstained with DAPI (mean nuclear intensity ± SEM). $^{**}P < 0.01$; ns $P > 0.05$ by ANOVA single factor.

F H2A ubiquitination levels in nuclear extracts (upper panel) and K63 ubiquitin chain formation on immunoprecipitated endogenous H2A (lower panel) from WT and *SirT7*$^{-/-}$ cells treated with IR (8 Gy) and 1-h chase analyzed by Western blot. Each WT and *SirT7*$^{-/-}$ sample is a pool of three independent cell lines.

G Analysis of H2A K15 and K13 ubiquitination after DNA damage in 293T cells transfected with a scramble or *SirT7* siRNA together with a Flag-tagged H2A vector in which the K118/K119 lysine has been inactivated (K118/K119R mutant) to eliminate polycomb-mediated ubiquitination. Cells were treated with IR (20 Gy) and after 1 h nuclear extracts were obtained and subjected to immunoprecipitation with an anti-Flag resin. Ubiquitination was inferred from mass shift of the H2A band measured by Western blot (lower panel).

Source data are available online for this figure.

© 2016 The Authors

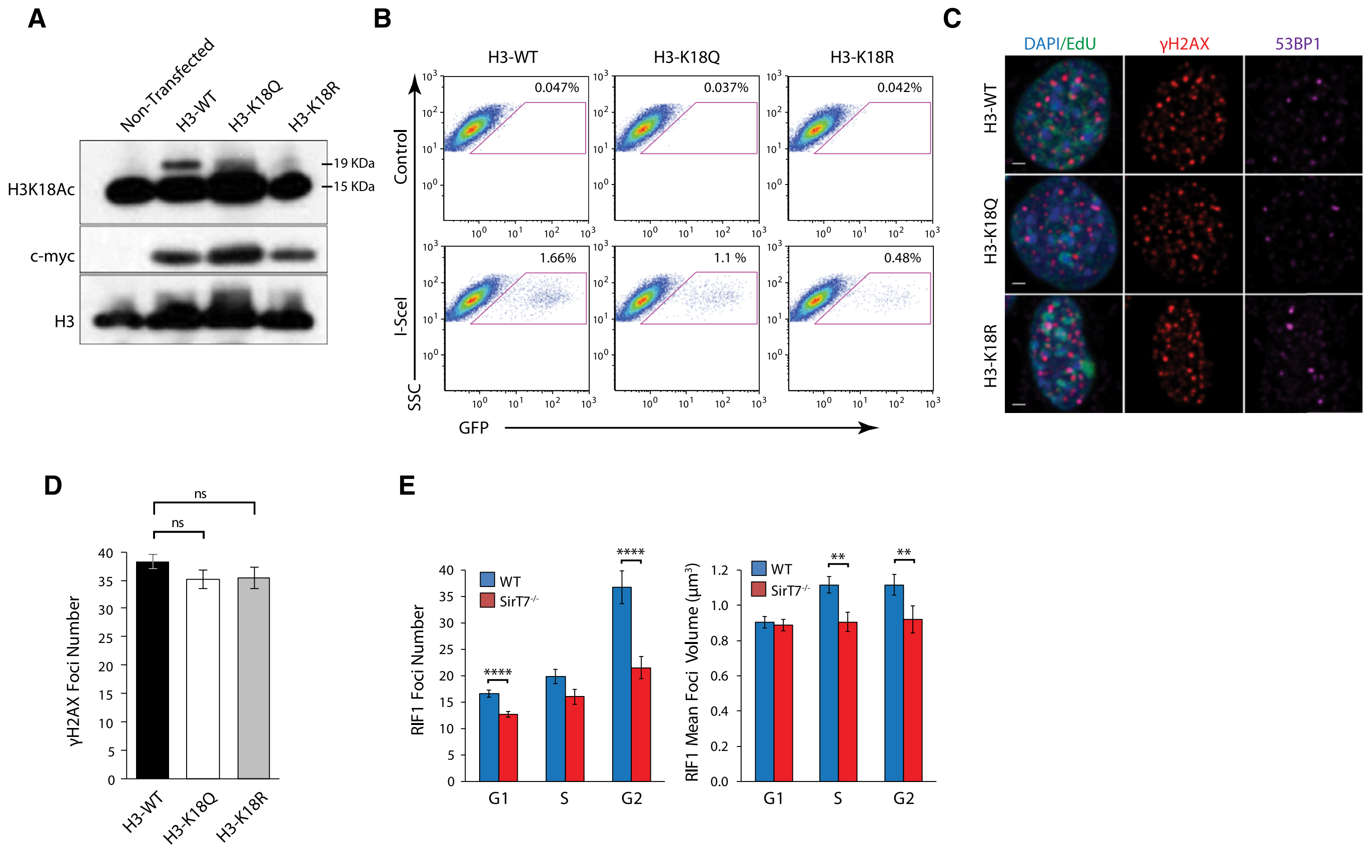

Figure EV5. H3K18 deacetylation is required for efficient 53BP1 recruitment to chromatin.

A Western blot showing H3K18Ac, c-myc, and total H3 expression in cells transfected with a Myc-tagged H3K18WT, H3K18R, and H3K18Q vector. Expected molecular weight for H3-Myc-tagged proteins and endogenous H3 is 19KDa and 15KDa, respectively. Even though ectopic expression is efficiently detected with an anti-Myc antibody, expression of recombinant histones is low compared with endogenous H3.

B Representative FACS analysis showing expression of the NHEJ-GFP reporter in HT1080 cells expressing H3-WT, H3-K18Q, or H3-K18R vectors.

C IF staining of NIH3T3 cells after IR (1 Gy) and 1-h chase. Cells were pulsed with EdU (green) 30 min prior to fixation, stained with antibodies against γH2AX (red) and 53BP1 (magenta), and counterstained with DAPI (blue). Representative images from cells in S-phase (EdU positive; scale bar 5 μm).

D IF quantitation of the number γH2AX foci after IR (1 Gy) and 1-h chase in NIH3T3 cells transfected with H3-WT-, H3-K18Q-, or H3-K18R-expressing vectors (mean ± SEM; 3 mice per genotype; ANOVA single factor).

E IF analysis of cells from WT and *SirT7*$^{-/-}$ fibroblasts during the cell cycle after IR (1 Gy) and 1-h chase. Cells were pulsed with EdU, stained with antibodies against γH2AX and RIF1, and then counterstained with DAPI (see Fig 7F for representative images). (Left) Quantitation of the number of RIF1 foci per nucleus, and (right) quantitation showing mean volume of RIF1 foci (mean ± SEM of > 30 cells per condition and cell cycle stage). *$P < 0.05$; **$P < 0.01$; ***$P < 0.001$; ****$P < 0.001$ by ANOVA single factor.

Source data are available online for this figure.

 © 2016 The Authors
