# Supplementary material for: SIRT7 promotes genome integrity and modulates non‐homologous end joining DNA repair
Source: EMBO J. 2016 May 25;35(14):1488–503. doi: 10.15252/embj.201593499 (PMC4884211; doi:10.15252/embj.201593499)

Expanded View Figure 4

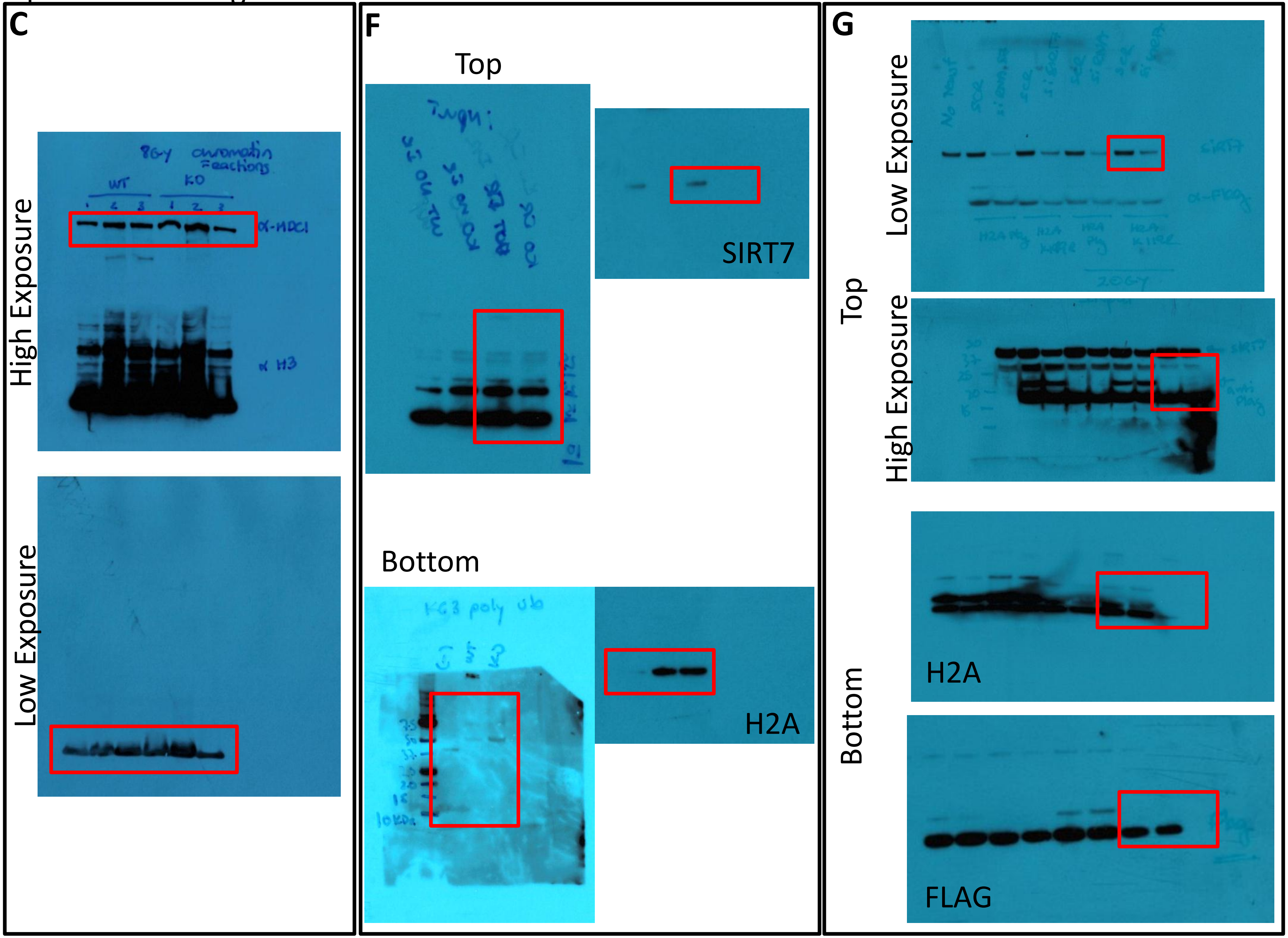

Supplement: Supplementary file 3 — Source Data for Expanded View [file EMBJ-35-1488-s003.zip › 93499_EV_Fig_Source_Data/Fig_EV4_SD/Fig_EV4_SD.pdf]
