# Supplementary figures and images for: SIRT7 promotes genome integrity and modulates non‐homologous end joining DNA repair
Source: EMBO J. 2016 May 25;35(14):1488–503. doi: 10.15252/embj.201593499 (PMC4884211; doi:10.15252/embj.201593499)

Expanded view Figure 3C

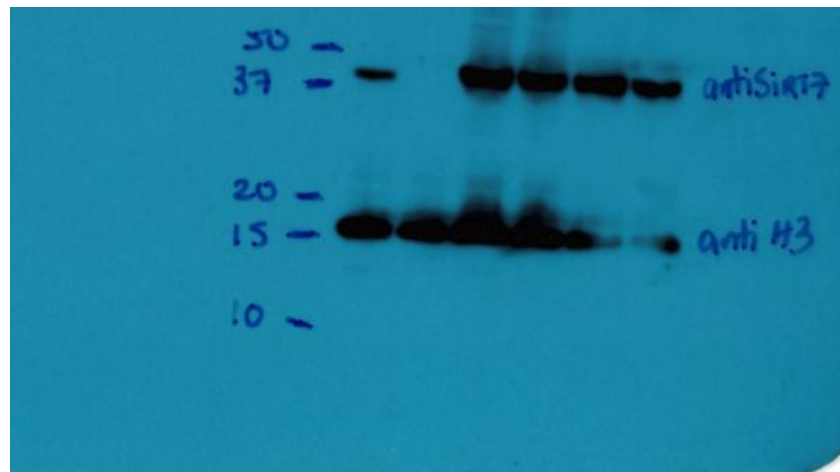

Supplement: Supplementary file 3 — Source Data for Expanded View [file EMBJ-35-1488-s003.zip › 93499_EV_Fig_Source_Data/Fig_EV3_SD/Fig_EV3_SD.pdf]

Expanded View Figure 5

A

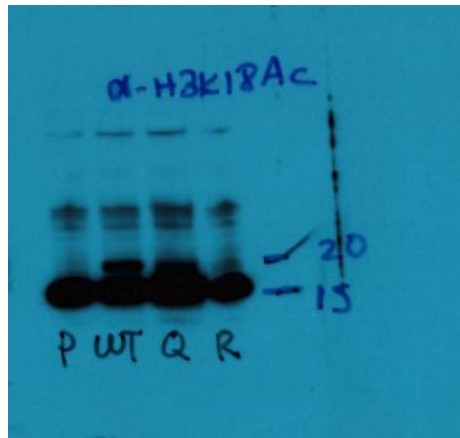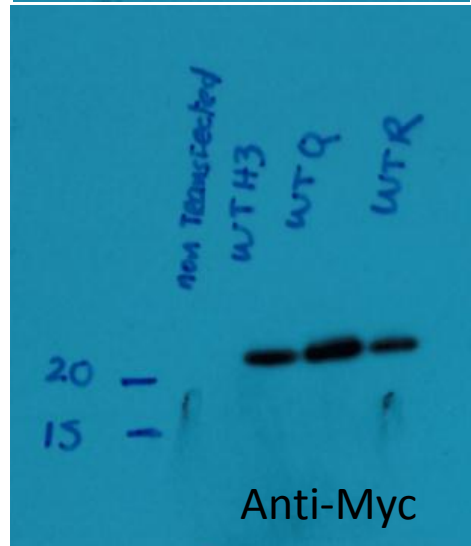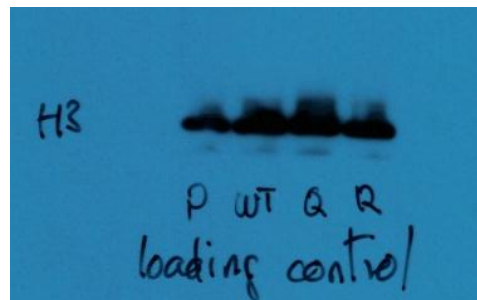

Supplement: Supplementary file 3 — Source Data for Expanded View [file EMBJ-35-1488-s003.zip › 93499_EV_Fig_Source_Data/Fig_EV5_SD/Fig_EV5_SD.pdf]

Figure 4B

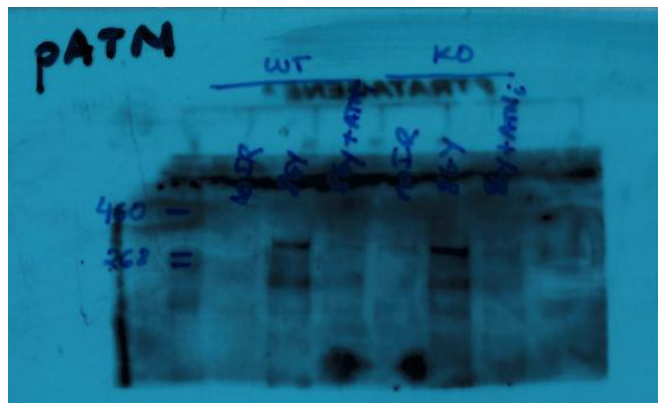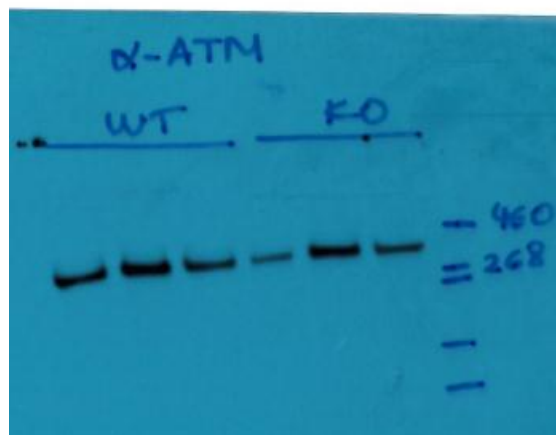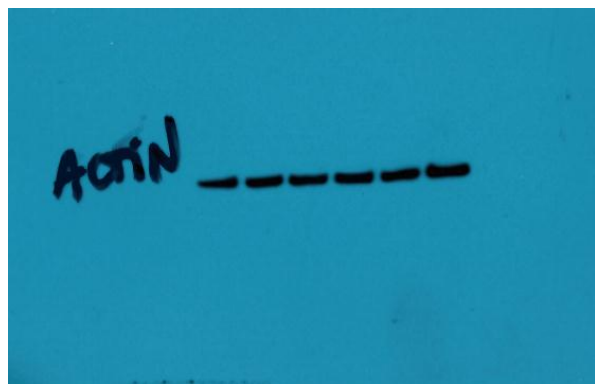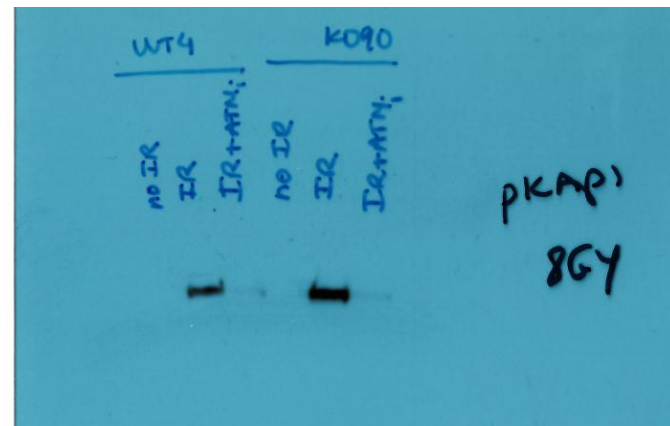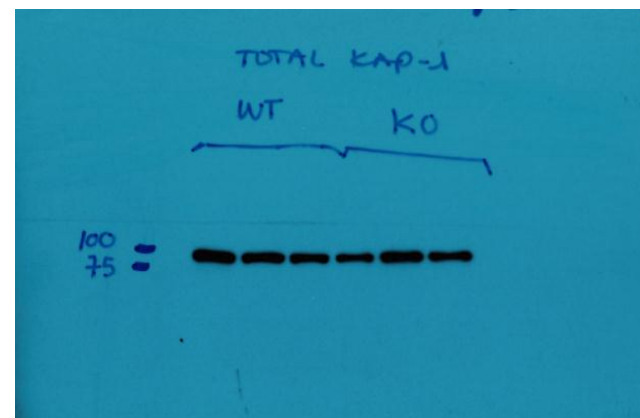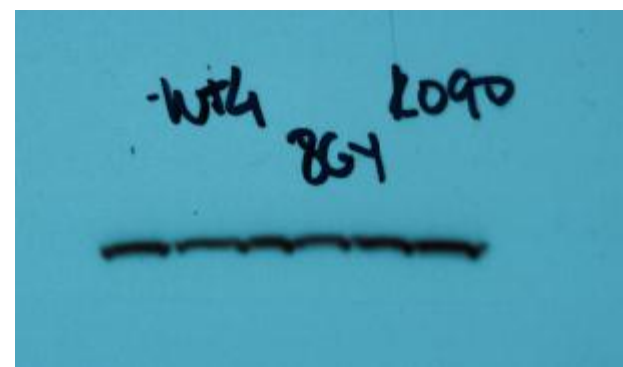

Supplement: Supplementary file 5 — Source Data for Figure 4 [file EMBJ-35-1488-s004.pdf]

Figure 5

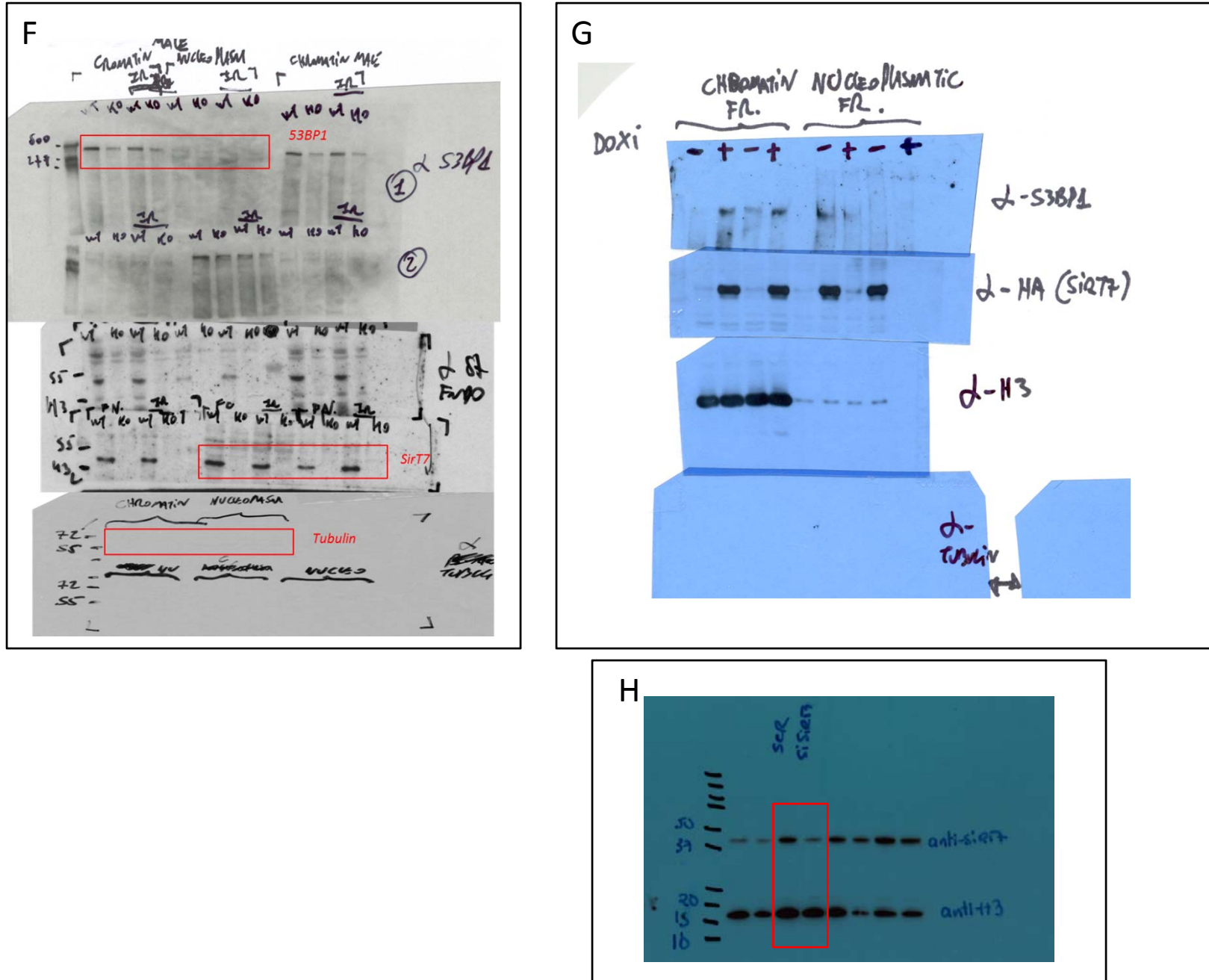

Supplement: Supplementary file 6 — Source Data for Figure 5 [file EMBJ-35-1488-s005.pdf]

Figure 7

E

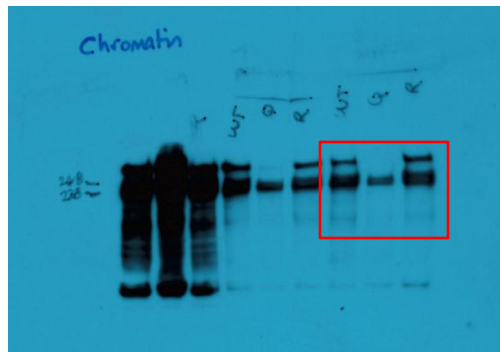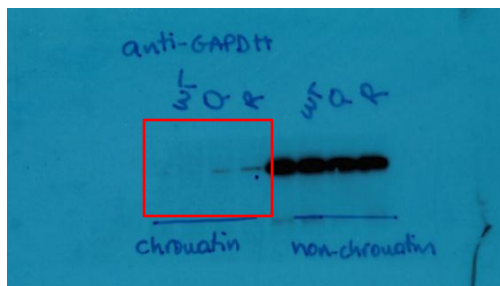

Anti-H3

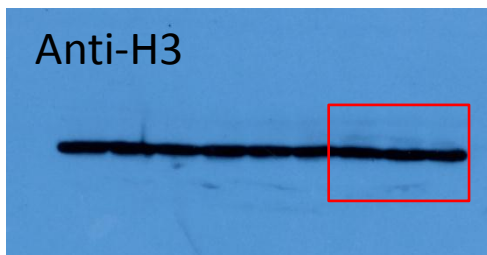

E

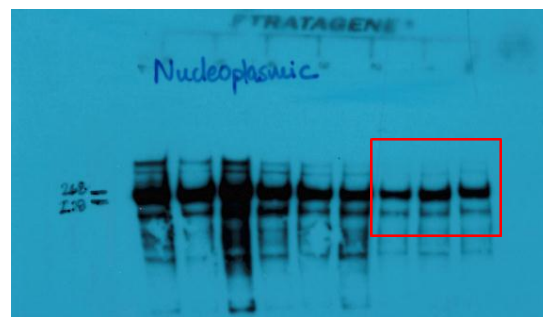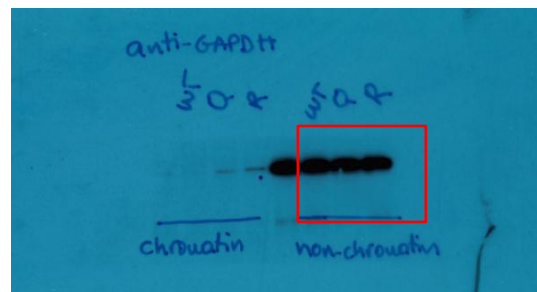

Anti-H3

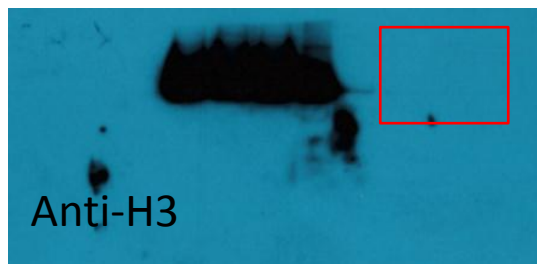

J

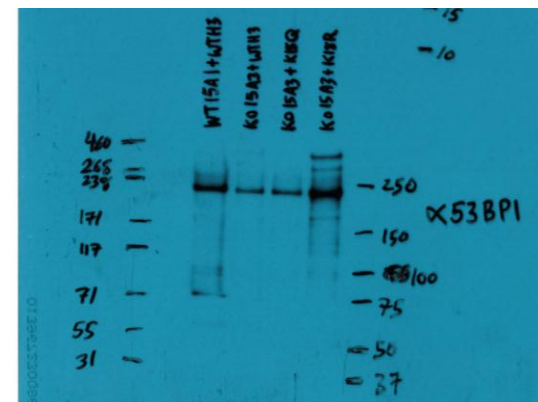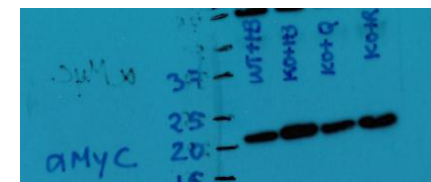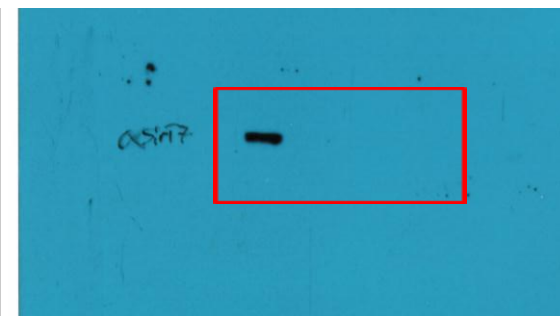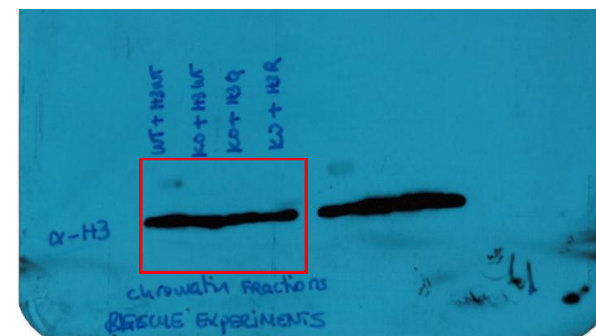

Supplement: Supplementary file 7 — Source Data for Figure 7 [file EMBJ-35-1488-s006.pdf]
